# The Older Adult Positivity Effect in Evaluations of Trustworthiness: Emotion Regulation or Cognitive Capacity?

**DOI:** 10.1371/journal.pone.0169823

**Published:** 2017-01-06

**Authors:** Leslie A. Zebrowitz, Jasmine Boshyan, Noreen Ward, Angela Gutchess, Nouchine Hadjikhani

**Affiliations:** 1 Department of Psychology, MS 062, Brandeis University, Waltham, MA, United States of America; 2 MGH/HST Athinoula A. Martinos Center for Biomedical Imaging, Harvard University, Charlestown, MA, United States of America; 3 Gillberg Neuropsychiatry Center, University of Gothenburg, Gothenburg, Sweden; Radboud Universiteit, NETHERLANDS

## Abstract

An older adult positivity effect, i.e., the tendency for older adults to favor positive over negative stimulus information more than do younger adults, has been previously shown in attention, memory, and evaluations. This effect has been attributed to greater emotion regulation in older adults. In the case of attention and memory, this explanation has been supported by some evidence that the older adult positivity effect is most pronounced for negative stimuli, which would motivate emotion regulation, and that it is reduced by cognitive load, which would impede emotion regulation. We investigated whether greater older adult positivity in the case of evaluative responses to faces is also enhanced for negative stimuli and attenuated by cognitive load, as an emotion regulation explanation would predict. In two studies, younger and older adults rated trustworthiness of faces that varied in valence both under low and high cognitive load, with the latter manipulated by a distracting backwards counting task. In Study 1, face valence was manipulated by attractiveness (low /disfigured faces, medium, high/fashion models’ faces). In Study 2, face valence was manipulated by trustworthiness (low, medium, high). Both studies revealed a significant older adult positivity effect. However, contrary to an emotion regulation account, this effect was not stronger for more negative faces, and cognitive load increased rather than decreased the rated trustworthiness of negatively valenced faces. Although inconsistent with emotion regulation, the latter effect is consistent with theory and research arguing that more cognitive resources are required to process negative stimuli, because they are more cognitively elaborated than positive ones. The finding that increased age and increased cognitive load both enhanced the positivity of trustworthy ratings suggests that the older adult positivity effect in evaluative ratings of faces may reflect age-related declines in cognitive capacity rather than increases in the regulation of negative emotions.

## Introduction

Considerable evidence has demonstrated an older adult (OA) positivity effect in attention and memory, although meta-analyses reveal that the effect is small [1–3]. Specifically, whereas younger adults (YA) tend to show preferential attention to and retrieval of negatively valenced faces, words, and pictures, OA do not show this negative bias and sometimes favor positively valenced stimuli [4–6]. There is also some evidence for a positivity effect in evaluative ratings of both neutral expression faces [7, 8] and emotion expression faces [9, 10]. These age differences may have significant social consequences given that first impressions from faces predict voting preferences and judicial decisions, among other socially important judgments [11]. The aims of the present research were to investigate whether OA positivity in evaluative ratings of neutral expression faces is moderated by cognitive load and by the valence of the faces, as manipulated by variations in facial attractiveness or trustworthiness. As discussed below, such moderation would support an emotion regulation explanation for this OA positivity effect.

Socio-emotional selectivity theory [12] provides a motivational mechanism for the OA positivity effects. According to this theory, a shorter future time perspective in OA yields greater concern with emotionally meaningful goals and greater motivation to engage in emotion regulation aimed at maintaining a positive mood. On this account, greater OA than YA positivity in attention, memory, and stimulus evaluation derives from less processing of negative stimuli and/or greater processing of positive stimuli in the interest of emotion regulation. Some research has tested this explanation by investigating the moderating effects of cognitive load, the mental effort being used in working memory. This research assumes that as cognitive load increases, the capacity for emotion regulation will decrease and the OA positivity effect will also decrease.

The studies testing whether the OA positivity effect in memory and attention is diminished when cognitive load reduces the opportunity for emotion regulation have produced mixed results [13–18]. One study [16] found that the typical effect shown with no distraction was reversed in a distraction condition, where negative pictures comprised a larger proportion of pictures recalled by OA than by YA and positive pictures comprised a larger proportion of pictures recalled by YA than by OA. Similarly, another study [15] found that the tendency for OA to attend less to negative (angry) faces and more to positive (happy) faces as compared with YA was reversed when people were distracted by a second task. OA also attended more than YA to positive (happy) faces given high attentional resources, but not when the task diminished those resources [14].

In contrast to the foregoing evidence that increased cognitive load interferes with the OA positivity effect in attention and memory, a pattern consistent with an emotion regulation explanation, other research provides conflicting evidence. In one study [17], there was an OA positivity effect in memory for positive vs. neutral or negative distracting words despite no apparent emotion regulation in attention to the words. Another study [13] further found that distraction yielded no change in the tendency for OA to focus less attention on negative than neutral pictures as compared with YA, suggesting that the OA positivity effect may not require the cognitive resources to regulate emotion. However, these authors noted that their distraction manipulation may have been weaker than those used in studies showing a reduction in the OA positivity effect. Therefore, the present investigation of age differences in the evaluation of faces manipulated cognitive load with a distractor task that required constant responding while viewing the visual stimuli, similar to the manipulation that yielded significant effects on age differences in attention and memory in previous research [15, 16].

In addition to mixed support for the claim that higher cognitive load reduces the OA positivity effect in attention and memory, there are also reasons to expect it to increase both YA and OA positivity in the case of evaluations. Processing of negative stimuli requires more cognitive resources because they are more cognitively elaborated than positive ones [19, 20]. Thus, interfering with such elaboration should decrease negative evaluations. Evidence consistent with this suggestion is provided by the finding that cognitive load increased self-enhancing self-descriptions in YA [21, 22], and it increased positivity of ratings of moral acts more than it decreased positivity for immoral acts [23]. Although the foregoing research investigated only YA, distraction may also serve to increase the positivity of OA evaluations by interfering with their capacity to process negative stimuli, rather than decreasing positivity by interfering with their emotion regulation capacity.

Like the moderating effects of cognitive load on the OA positivity effect, moderating effects of stimulus valence also bear on an emotion regulation explanation. Some evidence suggests that age differences in attention and memory are most pronounced for negatively valenced stimuli [24, 25], which are actually preferentially processed by YA [20, 26, 27]. This is consistent with the idea that the OA positivity effect reflects emotion regulation, which may be more engaged by negatively valenced stimuli. However, some research has failed to find stronger OA positivity for negatively valenced stimuli [28, 29]. Still other research found that the OA positivity effect in memory was shown for low arousing stimuli, regardless of valence, but not for high arousing stimuli [30]. In addition, greater OA positivity in facial expressions while viewing various stimuli was shown for neutrally valenced, but not positively or negatively valenced stimuli [31]. The reliability of a stronger OA positivity effect for negatively valenced stimuli is also uncertain in the domain of face evaluations. Although one study [8] found an OA positivity effect only for ratings of untrustworthy looking faces, this could have reflected age differences in responses to variations in face age and sex, which were not controlled across face valence, rather than reflecting responses to variations in trustworthiness per se. When face age and sex were controlled in another study [7], the OA positivity effect was equally strong for medium and low trustworthy faces.

Taken together the extant literature suggests that, compared with YA, OA show a positivity effect in attention, memory, and evaluation of stimuli that include emotionally evocative pictures [15, 16, 24], emotionally laden words [30], and faces varying in emotion expression or perceived traits [7, 8, 15, 27]. In addition, age differences may be most pronounced in responses to negative stimuli, although the data are mixed. Age differences also may be absent for highly arousing stimuli, as suggested by one study examining memory positivity [30]. Since many of the studies that found an OA positivity effect in attention and memory included both high and low arousal stimuli without comparing the effects [15, 16, 24, 25], it may be that the observed age differences also were due to the low arousal stimuli. Finally, research suggests that cognitive load can weaken the OA positivity effect in attention and memory, an effect attributed to the reduction of emotion regulation capacity. The present study investigated whether this effect will also be shown for OA positivity in evaluation of faces. Alternatively, it is possible that cognitive load will strengthen the positivity of evaluative responses for OA just as it has previously been shown to do for YA [21–23]. Two studies tested these competing hypotheses as well as the hypothesis that age differences in positivity would be most pronounced for the most negatively valenced faces. We also explored age differences in vision and cognitive function as possible mediators of age differences in positivity.

We assessed YA and OA ratings of the trustworthiness of faces that varied in valence (attractiveness or trustworthiness) both under low and high cognitive load. We did not focus on trustworthy ratings because of a particular interest in this specific trait. Rather, this focus was based on the fact that previous research has shown an OA positivity effect in trustworthy ratings [7, 8] that we expected to replicate, which was necessary to test our hypotheses regarding effects of valence and cognitive load. It should be noted that there is reason to expect effects on ratings of trustworthiness to generalize to other traits, because warmth/trustworthiness is one of two fundamental dimensions into which the traits ascribed to people can be organized. For our purposes, this dimension was preferable to a second independent dimension, power/competence [32, 33], because warmth/trustworthiness is marked by faster categorization with regard to valence [32–34].

In Study 1, face valence was manipulated by extreme variations in perceived attractiveness likely to elicit high arousal, and in Study 2 it was manipulated by variations in perceived trustworthiness. In both studies, cognitive load was manipulated by a distraction task. We predicted that 1) there would be an OA positivity effect, replicating previous evidence for more positive evaluative ratings by OA than YA. However, we anticipated that this effect may not be shown for faces that are also highly arousing. This result would show that high arousal stimuli are not vulnerable to the OA positivity effect in stimulus evaluations just as has been shown for memory [30]. An emotion regulation account of the OA positivity effect would further predict 2) a stronger OA positivity effect in ratings of negatively valenced than medium or positively valenced faces, and 3) a decrease in OA positivity under cognitive load in contrast to an increase for YA, with this age difference most pronounced for negatively valenced faces. In addition to testing these hypotheses, we performed exploratory analyses to determine whether age differences in visual or cognitive function mediated the differences in rating positivity.

## Materials and Methods: Study 1

### Participants

Twenty YA (10 men) aged 18 to 34 (M = 25.35, SD = 4.00) and 20 OA (10 men) aged 68 to 90 (M = 76.89, SD = 7.18) participated, and were paid $65 for completing the ratings as well as an fMRI study. OA were screened using the Mini-Mental State Examination [35] all scoring above 26 out of 30 (M = 29.37, SD = .96). Control measures revealed results consistent with other OA and YA samples previously tested in our lab, with OA performing worse on tests of visual acuity, contrast sensitivity, and speed (Pattern Comparison Test), and better on a vocabulary test. Although OA performed worse on some card sort task measures, they were no worse on perseverative errors, which is the measure of executive function (Table 1).

**Table 1:** Older and younger adult scores on control measures in Study 1.

Measure	Younger Adults		Older Adults			
	M	SD	M	SD	F-value	p-value
Snellen Visual Acuity (denominator)	14.11	4.91	27.50	9.59	29.08	< .001
Mars Letter Contrast Sensitivity (Mars Perceptrix, Chappaqua, NY)	1.66	.16	1.52	.20	6.00	.019
Benton Facial Recognition Test [36]	36.21	13.12	40.32	10.34	1.15	.291
Pattern Comparison Test [37]	41.47	7.83	29.95	6.18	25.39	< .001
Shipley Vocabulary Test [38]	31.95	3.36	35.84	2.95	14.43	.001
BCST Correct responses* [39]	38.30	2.10	31.39	8.78	11.68	.002
BCST Perseverative errors*	5.65	1.09	6.83	3.94	1.66	.205
BCST Non-perseverative errors*	4.05	1.76	9.94	9.33	7.70	.009
BCST Trials to complete first category*	9.45	1.36	11.94	9.02	1.50	.229

*BCST Berg Card Sort Task, a validated version of the Wisconsin Card Sort Task. N = 20 in each age group except that: data for one younger adult was missing for all control measures except for BCST; data for one older adult was missing for Mars, Benton, Pattern Comparison, and Shipley; and data for two older adults were missing for BCST.

### Face stimuli

Face valence was manipulated by variations in perceived attractiveness. Faces of 168 younger adults (84 men) were equally divided between high, medium, and low attractive categories as verified by pre-test ratings described below. High attractive faces (M = 5.21, SD = .06) were drawn from images of fashion models on agency websites. Medium attractive faces (M = 3.37, SD = .06) were drawn from publicly available face databases (Center for Vital Longevity Face Database: http://agingmind.utdallas.edu/facedb; FACES database: http://agingmind.utdallas.edu/facedb/view/neutralized-faces-by-natalie-ebner; Karolinska Directed Emotional Faces: http://www.emotionlab.se/resources/kdef). Low attractive faces (M = 1.43; SD = .06) were drawn from before images on plastic surgery websites and atlases of craniofacial anomalies.

We varied attractiveness so that we could include faces with an extreme negative valence to provide a sensitive test of the competing hypotheses that the OA positivity effect would be most pronounced for faces likely to engage emotion regulation or that it would be absent for highly arousing stimuli as shown in research on OA positivity in memory.

The 168 faces were selected based on pretest attractiveness ratings of 158 female faces and 158 male faces on 7-point scales with endpoints labeled not at all/very attractive. Ratings were provided by participants on Amazon Mechanical Turk (MTurk: https://requester.mturk.com/) who completed the study for payment of $1 per 15 minutes of participation. Thirty-four judges (23 males, mean age = 23.78, SD = 7.28 and 11 females, mean age = 36.27, SD = 12.31) rated female faces and 29 judges (15 males, mean age = 32.87, SD = 10.43 and 14 females, mean age = 34.21, SD = 8.31) rated male faces.

### Ratings

Faces were rated on 7-point scales with endpoints labeled ‘not at all trustworthy’ and ‘very trustworthy.’ Based on the well-documented attractiveness halo effect [11], we expected ratings of trustworthiness to track levels of attractiveness, although the finding that ‘ugly is bad’ is stronger than ‘beautiful is good’ provided reason to expect that low attractive faces might differ from average ones more than did high attractive faces [40].

### Cognitive load

Cognitive load was manipulated by variations in distraction. In the low distraction condition, a fixation cross appeared on the computer screen for 2 sec followed by a face for another 2 sec. After the face disappeared, a 7-point rating scale with endpoints labeled ‘not at all’ and ‘very’ trustworthy appeared and participants rated the face they had just seen. Once the rating was made, the next trial began (rating was self-paced). In the high distraction condition, a number appeared on the computer screen for 2 sec, which prompted participants to count aloud backwards from that number by threes. A face then appeared for 2 sec during which time participants continued to count until the face image was replaced with a 7-point rating scale with endpoints labeled ‘not at all’ and ‘very’ trustworthy on which participants rated the face they had just seen. An experimenter was present in the room to ensure that participants were performing the distraction task. He or she was seated at a desk on the opposite side of the experimental room and appeared engaged in a different task.

### Procedure

Face sex and distraction conditions were blocked (male distraction, male no distraction, female distraction, female no distraction) and the order of these blocks was counterbalanced across participants. Within each face sex/distraction block, there were 21 blocks of 4-faces (7 blocks each of attractive, medium, and unattractive faces). The faces were presented in a random order within each of these 21 face valence blocks, and the 21 blocks were counterbalanced so that faces of each valence followed faces of another valence with equal frequency. Faces were presented as 3” x 3” black and white images on a Dell Latitude E5440 laptop PC. The diagonal size of the anti-glare screen was 35.6 cm. Brightness was set to the maximum value possible and screen resolution was set to 1600 x 900.

### Ethics statement

Both studies were approved by IRB # 11010 at Brandeis University, and by IRB # 2013P000707 at Massachusetts General Hospital, and conducted according to the principles expressed in the Declaration of Helsinki. Participants signed a consent form prior to the experiment, and they were informed of their right to withdraw from the experiment at any time.

## Results: Study 1

We performed a 2 (Rater age:) x 2 (Rater sex) x 2 (Distraction) x 3 (Face Valence) repeated measures ANOVA, with face valence and distraction manipulated within subjects. Means and standard deviations are presented in Table 2. We also performed a planned interaction contrast [41] to test the specific prediction that the linear effect of face valence would differ by rater age. Another planned interaction contrast tested the 3-way interaction prediction that distraction would decrease rating positivity for OA, while increasing it for YA and that this effect would be most pronounced for negatively valenced faces. In addition, significant main effects were followed by simple effects comparisons to determine whether the overall effect held true in all conditions. Although we included rater sex in the analysis to account for any variance it might contribute, none of the effects of rater age, face valence, and distraction reported below were moderated by rater sex, and we do not report any other rater sex effects, as we had no predictions regarding this variable.

**Table 2:** Means and standard deviations of trustworthy ratings in Study 1.

	Negative				Medium				Positive			
	No Distraction		Distraction		No Distraction		Distraction		No Distraction		Distraction	
	M	SD	M	SD	M	SD	M	SD	M	SD	M	SD
Younger Adults	2.83	1.00	2.91	1.06	4.01	0.74	4.13	0.56	4.34	0.83	4.59	0.77
Older Adults	3.22	1.31	3.30	1.34	5.02	0.94	4.99	0.90	4.80	1.02	4.95	0.86

Note: Negative valence faces are unattractive, deformed faces; medium valence are average attractive faces; positive valence are high attractive faces of fashion models.

**Fig 1:**
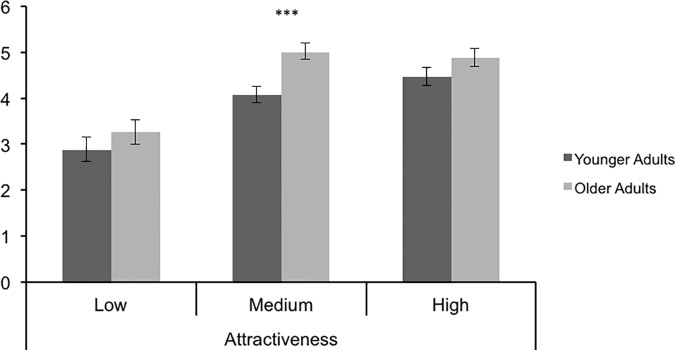
Trustworthy ratings by Face Valence and Rater Age in Study 1.

There was a strong effect of face valence, *F* (2,72) = 47.24, *p* < .001, η^2^ = .568, with significant differences between high (M = 4.67, SE = .133) or medium (M = 4.54, SE = .127), and low (M = 3.06, SE = .191) attractive faces, *p*s < .001, but no significant difference between high and medium attractive faces, *p* = .334. As predicted, OA gave higher trustworthy ratings (M = 4.38, SE = .156) to the faces than did YA (M = 3.80, SE = .156), *F*(1,36) = 6.89, *p* = .013, η^2^ = .161, thus replicating previously documented OA positivity effects in evaluative ratings of faces. However, contrary to the prediction that OA positivity would be strongest for the most negatively valenced, disfigured faces, there was no significant overall rater age x face valence effect, *F*(2,72) = 1.43, *p* = .247, η^2^ = .038. The interaction contrast analysis to more precisely test the prediction that the linear effect of face category would be stronger for one age group than the other was also not significant, *F* (1, 72) = .002, *p* = .964, d = .013, while the residual interaction effect was marginally significant, *F* (1,72) = 2.85, *p* = .096, d = .534. The simple effect comparisons of age differences within each level of attractiveness revealed that the overall OA positivity effect was significant for faces medium in attractiveness, *p* = .001, but not for those high, *p* = .129 or low in attractiveness, *p* = .315 (Fig 1). This pattern accounts for the marginally significant residual quadratic interaction effect.

There also was a significant main effect of distraction, with higher trustworthy ratings provided in the high distraction condition (M = 4.14, SE = .109) than the low distraction condition (M = 4.04, SE = .115), *F*(1,36) = 7.38, *p* = .010, η^2^ = .170, consistent with previous evidence that distraction increases evaluation positivity in YA. This effect was not moderated by rater age, *F*(1,36) = 1.12, *p* = .297, η^2^ = .03 or face valence, *F* (2,72) = 2.24, *p* = .114, η^2^ = .059. The 3-way interaction of rater age, face valence, and distraction was also not significant, *F* (2,72) = .403, *p* = .670, η^2^ = .011,and neither was the interaction contrast to test the specific prediction that distraction would decrease rating positivity for OA, while increasing it for YA, particularly for negatively valenced faces, *F*(1,72) = .28, *p* = .416, d = .166. The residual 3-way interaction effect also was not significant, *F* (1,72) = .53, *p* = .26, d = .230.

## Discussion: Study 1

Consistent with the attractiveness halo effect, low attractive, disfigured faces were rated as less trustworthy than average or high attractive faces. The fact that the latter two categories did not differ in perceived trustworthiness is consistent with some evidence that the halo effect reflects ‘ugly is bad’ more strongly than it reflects ‘beautiful is good’ [40]. Consistent with previous research on age differences in evaluations of faces [7, 8], there was an OA positivity effect, with OA rating the faces as more trustworthy than did YA. Given that the unattractive faces in the present study were markedly deformed, one would expect OA positivity to be most pronounced for these faces if it derived from the motivation to regulate negative emotions. However, the age x face interaction effect was not significant either in the omnibus test or in a planned contrast. Moreover, simple effects comparisons revealed that the age difference main effect in rating positivity was significant only for faces medium in attractiveness. Although inconsistent with emotion regulation, this result is consistent with the finding that emotional reactivity shown in facial expressions when viewing various stimuli revealed greater OA positivity for neutrally valenced, but not positively or negatively valenced stimuli [31]. The restriction of the OA positivity effect to medium attractive faces also is consistent with evidence that OA positivity in memory is shown for non-arousing but not for emotionally arousing words, be they positive or negative [30]. The images of disfigured individuals, who comprised our low attractive faces, and the fashion models, who comprised our high attractive faces, are arguably more arousing than images of ordinary individuals, who comprised our medium attractive faces. Even though high attractive faces were not rated as more trustworthy than the medium ones, the highly attractive faces may nevertheless have been more arousing. On this account, the restriction of the OA positivity effect to ratings of medium faces may be one instance of a tendency for that effect to be absent for strongly valenced or arousing stimuli. In the case of stimuli with a strong negative valence, this exception is inconsistent with an emotion regulation explanation for the OA positivity effect. Future research that directly assesses participants’ assessments of how arousing the faces are or their emotional responses to the faces would be useful to confirm this qualification to the OA positivity effect in evaluations.

Because we found no OA positivity effect in ratings of the disfigured faces, we could not test the emotion regulation prediction that cognitive load would decrease the tendency for OA to rate these faces more positively than do YA. Therefore, in Study 2 we used a set of faces that were less strongly valenced with the expectation that the negative faces in this set would yield an OA positivity effect in rated trustworthiness as it had in previous research [7, 8]. This would provide the opportunity to test the emotion regulation hypotheses that the OA positivity effect would be more pronounced for negatively valenced faces compared to neutral or positively valenced faces, and that it would be attenuated by cognitive load.

## Materials and Methods: Study 2

### Participants

Participants were 23 YA (12 men) aged 19 to 32 (M = 23.96, SD = 3.01) and 23 OA (12 men) aged 65 to 88 (M = 72.96, SD = 7.36). Participants were paid $65 for completing the ratings as well as participating in an fMRI study. OA were screened using the Mini-Mental State Examination [35] all scoring above 26 out of 30 (M = 29.44, SD = .99). Control measures replicated the age differences in Study 1, with OA performing worse than YA on tests of visual acuity, contrast sensitivity, and speed (Pattern Comparison Test), and better on a test of vocabulary (Table 3).

**Table 3:** Older and younger adult scores on control measures in Study 2.

Measure	Younger Adults		Older Adults			
	M	SD	M	SD	F-value	p-value
Snellen Visual Acuity (denominator)	14.69	5.45	27.50	10.00	17.11	< .001
Mars Letter Contrast Sensitivity (Mars Perceptrix, Chappaqua, NY)	1.73	.14	1.58	.22	6.17	.017
Benton Facial Recognition Test [36]	48.00	3.37	45.96	5.72	2.10	.154
Pattern Comparison Test [37]	41.59	8.40	30.17	5.80	28.38	< .001
Shipley Vocabulary Test [38]	32.62	2.89	35.70	2.62	13.73	.001
BCST Correct responses* [39]	38.43	2.41	31.48	7.60	17.52	< .001
BCST Perseverative errors*	5.70	1.15	6.96	3.62	2.53	.119
BCST Non-perseverative errors*	3.87	2.05	9.70	8.26	10.76	.002
BCST Trials to complete first category*	9.48	2.13	12.17	8.26	2.30	.137

*BCST Berg Card Sort Task, a validated version of the Wisconsin Card Sort Task. Ns = 23 in each age group except that: Snellen data were missing for 10 younger and 7 older participants; Mars data were missing for 3 younger and 1 older participants; Benton data were missing for 1 younger participant. Shipley data were missing for 2 younger participants.

### Face stimuli

Face valence was manipulated by variations in perceived trustworthiness rather than attractiveness, as in study 1. Photos of 180 women (90 older) were equally divided between high trustworthy (M = 4.78, SD = .22), medium trustworthy (M = 3.98, SD = .10), and low trustworthy (M = 3.12, SD = .29) categories, with each category significantly different from the rest, all *ps* < .001 These photos were selected from the same publicly available data bases used to select medium attractive faces in Study 1. The final 180 faces were selected based on pretest trustworthy ratings of 272 female faces obtained on Amazon Mechanical Turk (MTurk: https://requester.mturk.com/) from participants who completed the study for payment of $1 per 15 minutes of participation, using 7-point scales with endpoints labeled not at all/very trustworthy. A random order of older and younger female faces was rated by 46 judges (22 males, mean age = 32.71, SD = 9.09 and 24 females, mean age = 32.71, SD = 11.80).

### Cognitive load

Cognitive load was manipulated by the same distraction task used in Study 1.

### Procedure

Faces within each face age and valence were presented in a random order. Distraction was blocked, with the order of the blocks counterbalanced across participants.

## Results: Study 2

The means and standard deviations for trustworthy ratings in each condition are reported in Table 4. The analyses were identical to those performed in Study 1. As in Study 1, we do not report effects of Rater Sex as this variable did not moderate any of the predicted effects reported below.

**Table 4:** Means and standard deviations of trustworthy ratings in Study 2.

	Valence											
	Negative				Medium				Positive			
	No Distraction		Distraction		No Distraction		Distraction		No Distraction		Distraction	
	M	SD	M	SD	M	SD	M	SD	M	SD	M	SD
Younger Adults	3.06	0.74	3.25	0.73	3.76	0.76	4.01	0.72	4.56	0.81	4.62	0.82
Older Adults	3.97	1.04	4.19	1.05	4.70	0.94	4.77	0.96	5.36	0.85	5.27	0.87

Note: Negative valence faces are low trustworthy faces; medium valence are average trustworthy faces; positive valence are high trustworthy faces.

**Fig 2:**
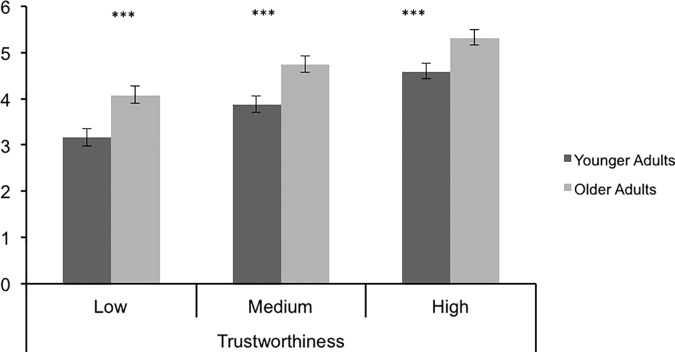
Trustworthy Ratings by Face Valence and Rater Age in Study 2.

A strong effect of face valence on trustworthy ratings, *F*(2, 84) = 212.57, *p* < .001, η^2^ = .835, revealed significant differences between high (M = 4.96, SE = .114), medium (M = 4.31, SE = .122), and low (M = 3.61, SE = .130) trustworthy faces, all *ps* < .001. As predicted, there also was a significant effect of rater age, with OA giving higher trustworthy ratings (M = 4.71, SE = .164) than did YA (M = 3.88, SE = .164), *F*(1,42) = 12.64, *p* = .001, η^2^ = .231. The non-significant omnibus interaction effect indicated that the OA positivity effect was not moderated by face valence, *F*(2, 84) = 1.15, *p* = .321, η^2^ = .027. The interaction contrast analysis performed to more precisely test the prediction that the linear effect of face category would be stronger for one age group than the other also was not significant, *F*(1, 84) = 1.12, *p* = .294, d = .311, and neither was the residual effect, *F*(1,84) = 1.19, *p* = .279, d = .321. The simple effect comparisons of age differences within each level of face trustworthiness revealed that the overall OA positivity effect was highly significant not only for medium valenced faces, as in Study 1, *p* = .001, but also for positively valenced, high trustworthy faces, *p* = .003, and negatively valenced, low trustworthy faces, *p* = .001 (Fig 2).

**Fig 3:**
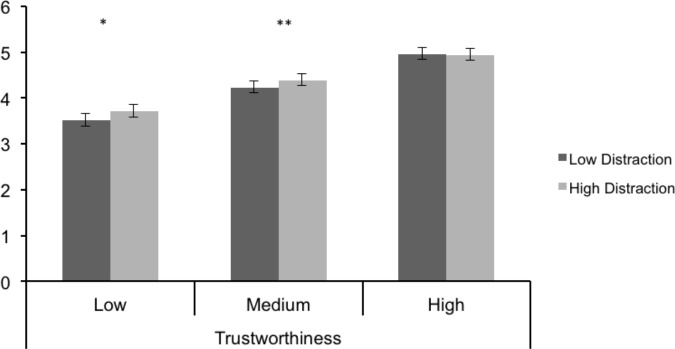
Trustworthy Ratings by Face Valence and Distraction in Study 2.

The main effect of distraction was not significant, *F*(1, 42) = 2.72, *p* = .106, η^2^ = .061, but there was a significant distraction x face valence effect, *F*(2, 84) = 10.08, *p* < .001. As shown in Fig 3, distraction increased the positivity of ratings for faces medium in trustworthiness, *p* = .041, and low in trustworthiness, *p* = .009, but not for those high in trustworthiness, *p* = .818. The distraction effect was not moderated by rater age, *F*(1, 42) = .680, *p* = .414, η^2^ = .016, and the OA positivity effect was significant under distraction and no distraction, both *ps* < .01. The rater age x face category x distraction effect also was not significant, *F*(2, 84) = 2.43, *p* = .094, η^2^ = .055, and neither was the interaction contrast to test the specific prediction that distraction would decrease rating positivity for OA, while increasing it for YA, particularly for negatively valenced faces, *F*(1, 84) = .58, *p* = .449, d = .224. However, the residual 3-way interaction effect was significant, *F*(1, 84) = 4.26, *p* = .042, d = .609. Rather than decreasing OA positivity for low trustworthy faces, as the emotion regulation explanation would predict, distraction marginally increased OA ratings of the trustworthiness of these faces, *p* = .051, with a similar trend for YA, *p =* .071. Distraction also significantly increased YA but not OA rating positivity for medium trustworthy faces, respective *ps* = .018 and .600, but had no effect on rating positivity for high trustworthy faces, *ps* = .576 and .377 for YA and OA, respectively.

Previous research had conducted post hoc analyses to determine whether age differences in vision or cognitive processes predicted differences in the positivity of trait ratings for representative samples of older and younger faces rated with no distraction [7]. Although the effects were only marginally significant, the only measure that both differed by age and also predicted greater OA positivity across several trait ratings was processing speed, as assessed by the pattern comparison test [37]. This can be explained if one assumes that slower processing on the pattern comparison task augurs greater cognitive load during the trait rating task. To determine whether the previous results could be replicated, we tested mediation of age differences in positivity of trustworthy ratings for the neutrally valenced faces (medium attractive and medium trustworthy), which had shown an OA positivity effect in both studies. This allowed us to collapse the data across Study 1 and 2 to increase power. We tested mediation not only by processing speed, but also by other control measures that differed by age (visual acuity, contrast sensitivity, BCST non-perseverative errors, vocabulary), and we examined mediation of age-related differences in positivity between age groups as well as within each age group.

The mediation analyses followed established guidelines [42] and were performed in SPSS utilizing PROCESS (version 2.15) add-on macro code [43]. Step 1 is the relationship between the putative causal variable (i.e. age) and the outcome variable (rating positivity). Necessary conditions for mediation are that the putative causal variable be significantly related to the mediator (Step 2) and that the mediator be significantly related to the outcome variable (i.e. rating positivity) with the causal variable controlled (Step 3) [42]. Finally, Step 4 is the change in the relationship between age and rating positivity when including the mediator in the model.

Analyses examining effects across age groups revealed that, compared to YA in Step 2, OA showed slower processing, *b* = -11.47, SE = 1.55, *t* = 7.42, poorer contrast sensitivity, *b* = -.17, SE = .03, *t* = 6.03, *p* < .001, more non-perseverative errors on the BCST, *b* = 5.85, SE = 1.35, *t* = 4.34, *p* < .001, higher vocabulary scores, *b* = 3.46, SE = .65, *t* = 5.37, *p* < .001, and poorer acuity on the Snellen test, *b* = 13.16, SE = 1.91, *t* = 6.88, *p* < .001 (note that higher Snellen scores signify worse acuity, as they denote the denominator on the Snellen Test). However, Step 3 was satisfied only for the visual acuity measure, such that higher Snellen scores/worse visual acuity were associated with lower rating positivity even with age controlled, *b* = -.04, SE = .01, *t* = 2.84, *p* = .006. Mediation analysis revealed that visual acuity acted as a suppresser of age differences in rating positivity, because the direct relationship between age and rating positivity in Step 1, *b* = .84, SE = .20, *t* = 4.20, *p* < .001, was even stronger at Step 4 after controlling for visual acuity, *b* = 1.30, SE = .25, *t* = 5.21, *p* < .001, and this increase was significant, *b* = -.46, Bootstrap SE = .20, Bootstrap 95% CI [-.905; -.092].

Analyses within age groups revealed that for YA neither Step 2, all *ps >* .283, nor Step 3, all *ps >* .095, was significant for any of the control variables. Among OA, processing speed alone satisfied the criteria for mediation. Not only was age a marginally significant predictor of rating positivity even within the truncated age range of OA (Step 1), *b* = .04, SE = .02, *t* = 1.93, *p* = .060, but also age predicted slower processing speed (Step 2), *b* = -.26, SE = .12, *t* = -2.23, *p* = .031, which in turn predicted greater rating positivity with age controlled (Step 3), *b* = -.052, SE = .024, *t* = 2.21, *p* = .033. Furthermore, processing speed was a significant mediator as shown by a non-significant relationship between age and rating positivity at Step 4 after controlling for processing speed, *b* = .022, SE = .02, *t* = 1.18, p = .247, with this decrease in the effect of age significant, *b* = .01, Bootstrap SE = .01, Bootstrap 95% CI [.001; .036].

## General Discussion

The results of the present study replicate and qualify previous evidence for an OA positivity effect in evaluative ratings of faces, and they argue against an emotion regulation explanation for this effect. Greater OA positivity was shown for trustworthy ratings of faces with positive, medium, or negative valence in Study 2 as well as for faces with a medium valence in Study 1. These effects are consistent with previous evidence for an OA positivity effect in trait ratings, including trustworthiness, for a representative sample of faces [7]. The pattern of greater OA positivity did not, however, extend to those faces with an extreme valence likely to elicit high arousal. These results are consistent with the finding that OA positivity in retrieval was shown for low, but not high arousal words [30], and it suggests that other research documenting the positivity effect in attention and memory may have done so only because low arousal stimuli were included in addition to high arousal stimuli [15, 16, 24, 25]. On the other hand, these results are not consistent with an emotion regulation explanation for OA positivity effects in evaluative ratings of faces, as emotion regulation processes should yield the strongest OA positivity effect for the extremely negative deformed faces in Study 1 and the more negatively valenced low trustworthy faces in Study 2, which we did not find.

In should be noted that Study 2 results contrast with the Castle et al. [8] finding of an OA positivity effect for untrustworthy, but not neutral or trustworthy faces. An important difference between the studies is that Castle et al. did not control the distribution of face age and sex across variations in face trustworthiness, whereas these potentially confounding variables were controlled both in Study 2 and in previous research that found an OA positivity effect in ratings of neutral as well as untrustworthy faces [7]. Thus, the Castle et al. findings may have reflected age differences in responses to face age and/or sex rather than a response to low trustworthiness per se.

Our results not only qualify previous evidence for an OA positivity effect in evaluative ratings of faces by demonstrating that the effect is not shown for faces that are extremely positive or negative, but also the distraction effects argue against the emotion regulation account that has been offered for OA positivity effects in attention and memory [13–17]. More specifically, rather than decreasing OA rating positivity, as predicted from an emotion regulation account, cognitive load increased OA rating positivity in both Studies 1 and 2. Most notably, it increased OA rating positivity for low trustworthy faces, which should have engaged the emotion regulation that distraction would disrupt. One might suggest that the faces used in our studies were not sufficiently negative to engage emotion regulation processes. However, this seems unlikely. Indeed, previous research has shown that compared with faces average or high in attractiveness, very ugly or disfigured faces, like those shown in Study 1, elicit greater amygdala activation [44, 45]. Similarly, a recent meta-analyses revealed that even normal faces that are low in trustworthiness, like those shown in Study 2, elicit greater amygdala activation than do high trustworthy faces [46].

While inconsistent with emotion regulation, the positive effect of distraction on trustworthy ratings is consistent with previous evidence that cognitive load increases the positivity of YA evaluations [21–23, 47], an effect replicated in the present study. In particular, the fact that distraction increased the positivity of trustworthy ratings for low but not high trustworthy faces in Study 2 is consistent with the argument that the reason cognitive load increases positivity is that more cognitive resources are needed to process negative stimuli because they are more cognitively elaborated than positive ones [19, 20]. Supporting the cognitive resources point is the recent argument that both OA and YA show more effortful processing when viewing negative than positive images, as indexed by pupil dilation [48]. Although those authors suggested that more effortful processing of negative images may have reflected an effort to suppress them from memory, it is also possible that it simply reflected the greater difficulty of processing negative information.

Although the prediction that distraction would decrease OA positivity is predicated on the argument made by previous researchers that the emotion regulation that fuels positivity is an effortful process that requires cognitive capacity [13–18], it could be argued that OA positivity is an automatic process that would not be compromised by distraction. Whereas an automatic process would argue against a decrease in positivity under distraction, it cannot explain why both OA and YA showed increased positivity under distraction. It also cannot explain why OA failed to show any positivity effect in evaluations of disfigured faces in Study 1 and showed no greater positivity in evaluations of low than high trustworthy faces in Study 2.

The fact that the positivity of trustworthy ratings can be augmented both by increased perceiver age and by increased cognitive load suggests that the OA positivity effect in evaluative ratings may be due to age-related declines in cognitive capacity rather than to increases in the regulation of negative emotions. Although age differences in processing speed, a proxy for cognitive capacity [40], did not mediate age group differences in rating positivity, some evidence to support a role for cognitive capacity in the OA positivity effect was provided by the finding that processing speed did mediate effects of age on rating positivity within the group of OA. Specifically, even within the truncated age range of this group, older participants rated the faces more positively and this effect was significantly reduced when processing speed was controlled, an effect that was consistent with post hoc results previously reported for evaluative ratings of different sets of faces [7]. Future research should employ additional measures of cognitive capacity to further investigate its contribution to age differences in the positivity of stimulus evaluations. It is possible that the previously demonstrated OA positivity effect in memory also may reflect age-related declines in cognitive capacity, as recent evidence showed that it was mediated by more effortful processing of the negative stimuli by OA than YA [46]. On the other hand, a negative relationship between cognitive capacity and OA memory positivity runs contrary to evidence that OA with higher scores on tests of cognitive control showed stronger positivity effects in memory tasks than those with lower scores [16]. Finally, although one might expect that the poorer vision of OA might impair sensitivity to negative facial cues, such as subtle resemblance of neutral expressions to negative emotions that influences trait ratings [49], controlling age differences in visual abilities did not eliminate the age differences in rating positivity. Rather, we found that age differences in visual acuity suppressed the OA positivity effect, which became stronger when the age differences in vision were statistically controlled.

It should be acknowledged that there are many possible explanations for an OA positivity effect in evaluative ratings of faces. One might suggest that it merely reflects a response bias, whereby OA have a greater tendency to rate faces at the high end of a scale. However, this explanation is not feasible, as greater OA positivity also was shown in previous research where OA rated faces lower on scales with endpoints labeled ‘not at all/very untrustworthy [7]. Additional evidence that the OA positivity effect in face ratings does not merely reflect rating scale use is the finding that these effects were paralleled by age differences in anterior insula activation [8]. One might also argue that the life experiences of OA, rather than emotion regulation, contribute to their greater positivity in evaluative ratings. However, this cannot explain why distraction increased the positivity of ratings for both OA and YA in the present study. One might also suggest that greater OA sympathy rather than greater emotion regulation could enhance OA positivity effect in ratings of disfigured faces. However, greater OA sympathy cannot explain the failure to find an OA positivity effect for these faces. Finally, although emotion regulation and cognitive capacity are not necessarily mutually exclusive explanations for OA positivity effects, the effect of distraction to increase OA positivity and the mediating effects of processing speed on OA positivity are both consistent with OA cognitive capacity limitations, but not emotion regulation.

## Conclusions

Our results replicate and qualify previous evidence for an OA positivity effect in evaluative ratings of faces. Most notably, we find that the effect is absent for faces that are disfigured or highly attractive, suggesting that highly arousing stimuli do not elicit age differences in evaluative positivity just as they may not elicit such differences in recall [30]. Future research that directly assesses participants’ emotional responses when evaluating faces or other stimuli would be useful to confirm this qualification to the OA positivity effect in evaluations. This qualification, coupled with the finding that cognitive load increased rather than decreased positivity for OA, argues against an emotion regulation explanation for the age differences in evaluative ratings. Specifically, high cognitive load would decrease emotion regulation capacity and should therefore decrease OA positivity if emotion regulation were the mechanism. Our results also rule out age differences in visual abilities as an explanation for greater OA positivity, as the poorer vision of OA suppressed rather than mediated the OA positivity effect. The parallel effects of cognitive load and age on positivity, coupled with evidence for a negative relationship between processing speed and positivity within the older adult group, suggests that lower cognitive capacity may contribute to the OA positivity effect in evaluations of faces. This possibility clearly merits further study with additional measures of cognitive capacity. There is also a need for additional research to evaluate the possibility that there is a dissociation in the mechanism that accounts for OA positivity effects in attention, and memory vs. evaluations, with emotion regulation playing a more significant role in the first two effects. Other possible mediating mechanisms also merit attention.

## Supporting Information

S1 File Raw data from Study 1. (XLSX)

S2 File Raw data from Study 2. (XLSX)

S3 File Data codes. (PDF)
