# Supplementary material for: The Older Adult Positivity Effect in Evaluations of Trustworthiness: Emotion Regulation or Cognitive Capacity?
Source: PLoS One. 2017 Jan 6;12(1):e0169823. doi: 10.1371/journal.pone.0169823 (PMC5218557; doi:10.1371/journal.pone.0169823)
Supplement: S3 File — (PDF) [file pone.0169823.s003.pdf]

Zebrowitz et al. PLOS ONE raw Data
Study 1 and Study 2 Codes:

AGE_CODE: Participant Age category (1 = younger adult/YA; 2 = older adult/OA)
SEX_CODE: Participant Sex (1 = male; 2 = female)
Age: Participant age in years
Avg: Trustworthy ratings of Medium Attractive faces (Study 1) or Medium Trustworthy faces (Study 2)
Att: Trustworthy ratings of High Attractive faces (Study 1)
Un: Trustworthy ratings of Low Attractive faces (Study 1)
All: Trustworthy Ratings averaged across male and female faces (Study 1) or younger and older faces (Study 2).
Fem: Trustworthy ratings of Female faces (Study 1)
Male: Trustworthy ratings of Male faces (Study 1)
High: Trustworthy ratings of High Trustworthy faces (Study 2)
Low: Trustworthy ratings of Low Trustworthy faces (Study 2)
D: Trustworthy ratings made with high distraction (Study 1 and 2)
ND: Trustworthy ratings made with low distraction (Study 1 and 2)
WCST: scores from Berg Card Sort Task (BCST; Piper et al., 2012).
MMSE: scores from Mini-mental state exam (administered only to OA)
Snellen: denominator from visual acuity test
MARS2: Letter contrast sensitivity with corrective lenses (Mars Perceptrix, Chappaqua, NY)
Benton: Facial recognition test; (Benton, Van Allen, Shamsher, & Levin, 1983).
Pattern: Pattern comparison Test (Salthouse, 1993).
Shipley: Shipley Vocabulary Test (Shipley, 1946).

Note: Trustworthy ratings made on 7-point scales with higher scores = more trustworthy
